# Body composition and muscle health changes after providing vascular imaging results in older adults: secondary analysis of a randomised controlled trial

**DOI:** 10.1007/s40520-026-03335-1

**Published:** 2026-02-11

**Authors:** Jack Dalla Via, Simone Radavelli-Bagatini, Marc Sim, Abadi K. Gebre, Catherine P. Bondonno, Kun Zhu, Shelby Mullin, John T. Schousboe, Richard J. Woodman, Markus P. Schlaich, Moira Sim, Pawel Szulc, Douglas P. Kiel, Wai H. Lim, Robin M. Daly, Jonathan M. Hodgson, Joshua R. Lewis

**Affiliations:** 1 Nutrition & Health Innovation Research Institute, School of Medical and Health Sciences, Edith Cowan University, Joondalup, WA Australia; 2 Institute for Physical Activity and Nutrition, School of Exercise and Nutrition Sciences, Deakin University, Geelong, VIC Australia; 3 Medical School, University of Western Australia, Perth, WA Australia; 4 Department of Endocrinology and Diabetes, Sir Charles Gairdner Hospital, Nedlands, WA Australia; 5 Park Nicollet Osteoporosis Center and HealthPartners Institute, and Division of Health Policy and Management, University of Minnesota, Minneapolis, MN USA; 6 Flinders Centre for Epidemiology and Biostatistics, Flinders University, Adelaide, SA Australia; 7 Dobney Hypertension Centre, Medical School, Royal Perth Hospital Research Foundation, Perth, WA Australia; 8 Department of Nephrology, Royal Perth Hospital, Perth, WA Australia; 9 Department of Cardiology, Royal Perth Hospital, Perth, WA Australia; 10 School of Medical and Health Sciences, Edith Cowan University, Joondalup, WA Australia; 11 INSERM UMR 1033, University of Lyon, Hospices Civils de Lyon, Lyon, France; 12 Hinda and Arthur Marcus Institute for Aging Research, Hebrew SeniorLife, Boston, MA USA; 13 Department of Medicine, Beth Israel Deaconess Medical Center, Harvard Medical School, Boston, MA USA; 14 Department of Renal Medicine, Sir Charles Gairdner Hospital, Perth, WA Australia

**Keywords:** Vascular calcification, Body composition, Metabolic health, Risk-reducing behaviours, Lifestyle

## Abstract

**Background:**

Provision of vascular imaging results has been investigated to prompt changes towards healthy lifestyle behaviours, but effects on body composition and muscle health are unknown.

**Aim:**

This secondary analysis of a 12-week parallel-group randomised controlled trial (RCT) aims to explore body composition and muscle health effects of providing healthy lifestyle education (Ed) with and without abdominal aortic calcification (AAC) results.

**Methods:**

A total of 240 Australian community-dwelling older men and women (mean ± SD age 68 ± 5 years; 58% female; 57.1% with evidence of AAC) were randomised to AAC + Ed (*n* = 121) or Control + Ed (*n* = 119). Linear mixed models were used to compare between-group changes in body composition (dual-energy X-ray absorptiometry), grip strength, and subjective physical function.

**Results:**

In total, 226 (94%) participants completed the trial. Provision of AAC results with lifestyle education provided no benefits to body composition, grip strength or physical function, compared to education alone. Exploratory analyses within the AAC + Ed group showed that those with evidence of AAC at baseline had greater declines in fat mass (net difference in change [95% CI] -0.6 [-1.0, -0.1] kg, *p* = 0.016) and visceral adipose tissue (-31 [-61, -1] g, *p* = 0.044) compared to those without evidence of AAC.

**Conclusions:**

Providing AAC results with healthy lifestyle education did not improve body composition or muscle health in older adults, compared to education alone. Provision of AAC results to those with evidence of AAC at baseline did improve total and visceral fat mass compared to those without evidence of AAC, but these findings require further investigation.

**Trial registration:**

Australian New Zealand Clinical Trial Registry (anzctr.org.au); registration number ACTRN12618001087246; registered 28/06/2018.

**Supplementary Information:**

The online version contains supplementary material available at 10.1007/s40520-026-03335-1.

## Introduction

Cardiovascular disease (CVD) and musculoskeletal conditions are among the leading causes of disease burden for older adults in Australia [1] and globally [2]. Overweight and obesity in particular is the leading contributor to non-fatal disease burden [1]. The nexus between cardiovascular and musculoskeletal systems is becoming increasingly evident, and likely related to shared risk factors (e.g. increasing age, low physical activity, poor diet) and common pathophysiological mechanisms (e.g. inflammation) [3–5]. Importantly, there are many common approaches recommended to manage both cardiovascular and musculoskeletal health in older adults, such as regular exercise and maintaining a healthy diet [6–8]. Therefore, simultaneously assessing cardiovascular health, body composition and musculoskeletal health may help to direct preventative management approaches and reduce their disease burden.

In addition to bone density, dual-energy X-ray absorptiometry (DXA) can provide measures of body composition, including total and regional fat and lean mass [9], making it an important tool to evaluate and monitor musculoskeletal health. Lateral spine images (LSI) obtained using DXA can also assess abdominal aortic calcification (AAC), which is a measure of structural vascular disease that is closely related to vascular disease in other arteries and predictive of CVD events and mortality [10, 11]. Therefore, DXA may be particularly useful to concurrently screen for subclinical CVD and poor body composition, in addition to low bone density, in middle-aged and older adults.

Beyond screening for elevated risk of CVD events, vascular disease imaging has also been investigated as a tool to promote risk-reducing or preventative lifestyle behaviour change. This is based on the notion that behaviour change is more likely with greater perceived susceptibility and severity of an adverse event [12]. Observational studies and randomised controlled trials (RCTs) investigating provision of vascular imaging results have predominantly focused on cardiovascular health, showing mixed results for improving cardiovascular risk-reducing lifestyle behaviours such as improved diet and increased physical activity [13]. Mixed effects on body weight, body mass index (BMI) and waist circumference have also been observed in some studies [13], but no known study has reported the effects of providing vascular imaging results on DXA-derived body composition or muscle health outcomes. The modification of diet, exercise and lifestyle (MODEL) RCT investigated the influence of providing AAC results on fruit and vegetable intake, physical activity and other CVD risk factors in older Australian adults [14, 15]. While AAC provision improved some CVD risk factors, it had no effect on the primary outcome of fruit and vegetable intake or secondary outcomes such as diet quality, or physical activity over 12 weeks [15].

The aim of this secondary analysis from the MODEL RCT was to investigate whether providing AAC results with healthy lifestyle educational resources led to improvements in body composition, muscle strength and physical function in older Australian men and women, compared to healthy lifestyle educational resources alone. Our a priori hypothesis for the MODEL RCT was that the provision of AAC would improve body composition and muscle health.

## Material & methods

### Study design

As reported previously [15], the MODEL study was a 12-week parallel-group RCT involving Western Australian men and women aged 60 to 80 years. The primary aim of the study was to examine whether provision of AAC results with diet and physical activity education improves fruit and vegetable intake, compared to diet and physical activity education alone. The MODEL study originally included a pre-planned observational component, in which all participants (AAC + healthy lifestyle education [Ed] and Control + Ed who received their AAC results at 12 weeks) would repeat the study assessments at two years follow-up. This was designed to allow comparisons of two-year changes in outcomes between participants with and without evidence of AAC at baseline. Due to the impact of COVID on the original study budget, this was no longer viable to complete. Therefore, in addition to a secondary data analyses of intervention effects, this study includes 12-week effects (i.e. baseline to 12 weeks within the AAC + Ed group) on the observational outcomes that were originally planned for two years. Consequently, bone density data are not presented as we are unlikely to observe any true physiological changes given the typical bone remodelling cycle takes approximately six months. The study methods have been reported in detail previously [14, 15], with a brief overview of the methods relevant to the current study aims provided below.

Participants were randomised (1:1 ratio) to receive their AAC results with educational resources (AAC + Ed group) or to receive educational resources alone (Control + Ed group). The allocation sequence and block size were generated by a statistician (RW). The final study (redesigned after termination in March 2020 due to COVID-19) was approved in August 2020 by the Edith Cowan University Human Research Ethics Committee (Project number: 20513 HODGSON) and registered with the Australian New Zealand Clinical Trial Registry (ACTRN12618001087246). All participants provided informed consent prior to their inclusion in the study.

### Participants

Community-dwelling men and women from Perth, Western Australia were recruited via newspaper advertisements. Participants were deemed eligible, via telephone screening, if they were aged 60 to 80 years, could attend in person clinic visits and complete electronic questionnaires, and had a mobile phone. Eligible participants attended a baseline clinic visit at the Sir Charles Gairdner Hospital Bone Density Unit, where they provided written consent prior to completing baseline assessments. Participants were enrolled in three waves comprising 75–90 participants per wave. The 12-week intervention period was conducted between September 2020 and April 2021 for participants in wave 1, May to September 2021 for participants in wave 2, and October 2021 to May 2022 for participants in wave 3.

### Intervention

All participants were informed of their group allocation via email approximately 2 weeks after their baseline visit. In addition to group allocation, emails to both groups included a link to a ~ 12-minute video, supporting e-booklet, and a frequently asked questions document. The video was developed by the research team and contained information on CVD [16] (the video for the AAC + Ed group also included an explanation of AAC and its link with CVD risk) and dietary and physical activity recommendations [17–19] related to the three key goals of the study: (1) increase fresh fruit intake by at least 1 serve/d (150 g/d) and increase vegetable intake by at least 1 serve/d (75 g/d); (2) improve other aspects of diet (i.e., reduce intake of salt, sugar, processed foods and increase wholegrains and nuts); and (3) increase physical activity and reduce sitting time. For participants allocated to the AAC + Ed group, the email also contained a letter with their individual AAC result. Participants in the Control + Ed group received their AAC results at the end of the 12-week study period. As presented previously (see Fig. 2 in [15]), the AAC results letter included the participants own lateral spine image, along with a diagram representing lumbar spine vertebrae 1–4 and the anterior and posterior abdominal aortic wall in this region and a brief explanation of the results. For participants with evidence of AAC, arrows on the lateral spine image and red lines on the diagram were added to illustrate where calcification was identified, and a red dot was used to indicate evidence of advanced blood vessel disease. For participants without evidence of AAC, the LSI and diagram were left blank (i.e. no arrows, markings added), and a green dot was used to indicate no evidence of advanced blood vessel disease. The AAC letter, along with an information letter about AAC, was also sent to the general practitioner of each AAC + Ed participant at baseline. All participants received a phone call on the same day they received their results, which focused on discussing the AAC result for participants in the AAC + Ed group, and to go over the goals of the study and discuss diet and physical activity with participants in both groups.

### Outcome measures

All outcome measures were assessed at baseline and 12 weeks. Body weight was measured using digital scales to the nearest 0.1 kg, and height measured using a wall-mounted stadiometer to the nearest 0.1 cm, both with participants wearing lightweight clothes and no shoes. DXA scans were performed at Gairdner Bone Densitometry Services, Sir Charles Gairdner Hospital, Perth, using a Hologic Horizon A densitometer (Hologic Inc., Bedford, MA, USA). Each participant completed scans of the whole body, lateral spine, lumbar spine and proximal femur. AAC was assessed on the LSI by an expert in densitometric imaging (JTS) and scored using an established semi-quantitative scoring system (ranging from 0 to 24; AAC24) [11, 20–22]. Briefly, an AAC24 score indicates the proportion of the anterior and posterior walls of the abdominal aorta (at the L1-L4 levels) where calcification is observed, such that a higher score suggests more extensive calcification. Total body (excluding the head) lean soft tissue and fat mass, as well as appendicular lean soft tissue mass (sum of arms and legs) were obtained from total body scans. Visceral adipose tissue mass was estimated using Hologic’s InnerCore™ Visceral Fat Assessment. Visceral fat is calculated as subcutaneous fat subtracted from total fat, assessed in the abdominal region of the total body scan, which is a ~5 cm region extending superiorly from the iliac crest [9]. Grip strength was assessed as the highest of three attempts with the dominant hand using a handheld dynamometer (Jamar Hydraulic Hand Grip Dynamometer, Nottinghamshire, UK). Subjective measures of physical health were derived from the 36-item Short Form Health Survey (SF-36) [23]. Five scales related to physical health were extracted: Physical functioning (10 items; 3-point Likert scale), role limitations due to physical health (4 items; Yes or No), energy/fatigue (4 items; 6-point Likert scale), pain (2 items; 5- or 6-point Likert scale), and general health (5 items; 5-point Likert scale). Each item is scored from 0 (lowest possible score) to 100 (highest possible score), such that scores represent the proportion of the total possible score. The average score of items in each scale was then calculated, with a higher score representing a more favourable health status. Dietary intake was assessed using the validated Dietary Questionnaire for Epidemiological Studies Version 3.2 (DQES v3.2), which is a food frequency questionnaire developed by the Cancer Council of Victoria [24]. While this questionnaire is designed to capture intake over 12 months, participants were asked to report their intake based only on the previous 12 weeks to better reflect the study timeframe. Totel energy and macronutrient (protein, fat, carbohydrate) intake were extracted, then divided by body weight to determine relative intakes. The Community Healthy Activities Model Program for Seniors (CHAMPS) questionnaire was used to estimate physical activity levels, with energy expenditure for total and moderate-to-vigorous physical activity calculated as per the CHAMPS scoring instructions [25], then converted to kJ/day.

### Statistical analysis

Data were analysed using Stata statistical software (Version 18.0; Stata, College Station, TX, USA) with an intention-to-treat approach including all participants randomised. Baseline and 12-week outcome data were assessed with descriptive analyses. Between-group differences for the changes over 12 weeks for each outcome were analysed using mixed effects regression with random effects for participants, fixed effects for group and time (baseline and 12 weeks), and an interaction between group and time. Within group changes for each group in each outcome from baseline to 12 weeks were determined using post-estimation margins following the mixed effects regression models. Outcome variables were tested for normality and log-transformed if necessary. Also, level 1 and level 2 residuals from the linear mixed models were assessed for normality. As the planned observational component of the MODEL study was not able to be completed, exploratory analyses were conducted within the AAC + Ed group to investigate whether presence (AAC24 ≥ 1) or absence (AAC24 = 0) of AAC at baseline, with the provision of education, influenced outcomes over the 12-week RCT period. To determine whether higher levels of disease led to greater changes in body composition, muscle strength, and physical function outcomes, associations between baseline AAC24 score and change in outcomes were analysed using Spearman Rho (ρ) as AAC24 is a restricted ordinal variable with a range between 0 and 24. Within the AAC + Ed group, changes in all outcomes were compared between participants with and without evidence of AAC using independent t-tests. Statistical significance was set at *P* < 0.05 for all analyses.

## Results

The results for the primary outcomes of the MODEL study – objective (plasma carotenoid levels) and subjective (food frequency questionnaire) fruit and vegetable intake, along with major secondary cardiovascular health outcomes have been reported previously [15].

### Participants

As reported previously [15], of the 245 participants recruited from September 2020 to May 2022, 240 were randomised. Of these, 226 (94%) completed the 12-week trial, with similar attrition in each group (AAC + Ed 5.0%; Control + Ed 7.2%). Participant baseline characteristics have been reported in detail [15]. Briefly, the mean ± SD age was 67.8 ± 5.0 years (AAC + Ed 68.0 ± 4.9 years; Control + Ed 67.5 ± 5.1 years), 57.5% were females (AAC + Ed 57.8%; Control + Ed 57.1%), and 57.1% had evidence of AAC (AAC + Ed 57.8%; Control + Ed 56.3%) with a median (IQR) AAC24 score of 2 (1, 3).

### Body composition

Provision of AAC had no effect on any body composition outcome (Table 1). BMI and total fat mass declined significantly within Control + Ed only, while appendicular lean soft tissue mass and visceral adipose tissue declined significantly within AAC + Ed only. Total lean soft tissue mass declined significantly in both groups, and body fat percentage did not change in either group.

**Table 1:** Within and between-group changes in body composition, muscle strength and subjective physical function outcomes in the AAC + Ed and Control + Ed groups

	AAC+Ed group		Control+Ed group			
	*n*	Mean ± SD or (95% CI)	*n*	Mean ± SD or (95% CI)	Mean net difference in change (95% CI) ^a^	*p*-value Interaction ^b^
**Body composition**						
BMI (kg/m^2^)						
Baseline	121	26.7 ± 4.7	119	27.7 ± 4.9		
12 weeks	115	26.5 ± 4.6	112	27.5 ± 4.9		
Within-group change ^c^	115	−0.1 (−0.3, 0.04)	112	**−0.2 (−0.3**, −0.03)*	0.1 (−0.1, 0.3)	0.529
Total fat mass ^d^ (kg)						
Baseline	121	23.0 ± 8.3	119	24.3 ± 8.8		
12 weeks	114	22.6 ± 8.2	110	24.0 ± 9.0		
Within-group change ^c^	114	−0.2 (−0.5, 0.04)	110	**−0.3 (−0.6**, −0.01)*	0.1 (−0.3, 0.4)	0.757
Body fat percentage ^d^ (%)						
Baseline	121	31.5 ± 8.1	119	32.5 ± 8.7		
12 weeks	114	31.5 ± 8.0	110	32.2 ± 8.8		
Within-group change ^c^	114	−0.1 (−0.3, 0.2)	110	−0.2 (−0.4, 0.1)	0.1 (−0.2, 0.5)	0.561
Total lean soft tissue mass ^c^ (kg)						
Baseline	121	47.5 ± 11.3	119	48.3 ± 11.2		
12 weeks	114	46.9 ± 11.3	110	48.1 ± 11.0		
Within-group change ^c^	114	**−0.4 (−0.7**, −0.2)***	110	**−0.3 (−0.5**, −0.1)*	−0.1 (−0.5, 0.2)	0.400
Appendicular lean soft tissue mass (kg)						
Baseline	121	21.1 ± 5.7	119	21.2 ± 5.3		
12 weeks	114	20.8 ± 5.6	110	21.1 ± 5.2		
Within-group change ^c^	114	**−0.2 (−0.4**, −0.1)***	110	−0.1 (−0.3, 0.01)	−0.1 (−0.3, 0.1)	0.295
Visceral adipose tissue mass (g)						
Baseline	121	591 ± 291	119	623 ± 287		
12 weeks	114	573 ± 279	110	614 ± 297		
Within-group change ^c^	114	**−18 (−35**, −1)*	110	−13 (−30, 5)	−5 (−29, 19)	0.685
**Muscle strength**						
Grip strength (kg)						
Baseline	121	34.4 ± 11.3	119	34.5 ± 10.9		
12-weeks	115	34.2 ± 11.0	111	34.5 ± 11.7		
Within-group change ^c^	115	−0.2 (−0.8, 0.4)	111	0.03 (−0.6, 0.7)	-−0.2 (−1.1, 0.6)	0.595
**Subjective physical function (SF-36 questionnaire)**						
Physical functioning						
Baseline	121	83.2 ± 16.0	119	80.7 ± 20.6		
12-weeks	115	83.8 ± 18.2	112	84.2 ± 18.1		
Within-group change ^c^	115	0.5 (−2.3, 3.3)	112	2.6 (−0.2, 5.5)	−2.1 (−6.1, 1.9)	0.295
Role limitations due to physical health						
Baseline	121	73.6 ± 37.4	119	79.4 ± 34.8		
12-weeks	115	76.7 ± 36.1	112	77.2 ± 36.8		
Within-group change ^c^	115	3.0 (−3.5, 9.5)	112	−2.2 (−8.8, 4.4)	5.2 (−4.1, 14.5)	0.271
Energy/fatigue						
Baseline	120	66.6 ± 17.5	119	67.9 ± 20.1		
12-weeks	115	67.1 ± 19.0	112	68.2 ± 20.6		
Within-group change ^c^	115	0.8 (−1.4, 3.0)	112	−0.1 (−2.3, 2.1)	0.9 (−2.2, 4.0)	0.571
Pain						
Baseline	120	76.0 ± 19.7	119	75.9 ± 19.8		
12-weeks	115	77.1 ± 21.6	112	77.1 ± 22.0		
Within-group change ^c^	115	1.1 (−2.0, 4.2)	112	0.6 (−2.5, 3.8)	0.5 (−3.9, 4.9)	0.834
General health						
Baseline	121	71.0 ± 17.1	119	70.5 ± 17.4		
12-weeks	115	74.0 ± 15.7	112	71.6 ± 17.1		
Within-group change ^c^	115	**2.7 (0.4**, 5.0)*	112	0.9 (−1.4, 3.2)	1.8 (−1.4, 5.1)	0.275

Baseline and 12-week data were estimated using descriptive statistics in Stata, with mean ± SD (or 95%CI) reported. Within-group changes and mean between-group differences for the change over time were estimated using linear mixed models with values representing means and 95%CI. ^a^ Difference in change between AAC + Ed vs. Control + Ed groups. ^b^ p-values for the interaction between treatment group and timepoint from linear mixed models. ^c^ Within-group change from post-estimation margins. ^d^ Head excluded. ^*^ Indicate statistical significance within-group changes from baseline to 12 weeks (^***^p < 0.001, ^**^p < 0.01 and ^*^p < 0.05). Bold p-values indicate statistically significant differences. Abbreviations: AAC, abdominal aortic calcification; Ed, healthy lifestyle education; BMI, body mass index

### Muscle strength and physical function

AAC + Ed had no effect on grip strength or subjective physical function (physical SF-36 domains) compared to Control + Ed. Scores in the general health domain of the SF-36 increased significantly within the AAC + Ed, but there were no other within-group changes in either group (Table 1).

### Diet and physical activity

In the current analyses, there were no group differences for changes in body weight-adjusted total energy or macronutrient intake, or on estimated energy expenditure from total or moderate-vigorous physical activity (Table 2).

**Table 2:** Within and between-group changes in diet and physical activity outcomes in the AAC + Ed and Control + Ed groups

	AAC+Ed group		Control+Ed group			
	*n*	Mean ± SD or (95% CI)	*n*	Mean ± SD or (95% CI)	Mean net difference in change (95% CI) ^a^	*p*-value Interaction ^b^
**Diet**						
Relative energy intake (kJ/kg BW/day)						
Baseline	120	119.7 ± 36.8	117	115.0 ± 33.4		
12 weeks	115	117.0 ± 39.0	112	112.1 ± 33.6		
Within-group change ^c^	115	−2.2 (−6.5, 2.1)	112	−3.1 (−7.5, 1.3)	0.9 (−5.2, 7.1)	0.765
Relative protein intake (g/kg BW/day)						
Baseline	120	1.27 ± 0.43	117	1.17 ± 0.40		
12 weeks	115	1.25 ± 0.42	112	1.15 ± 0.35		
Within-group change ^c^	115	−0.01 (−0.06, 0.04)	112	−0.02 (−0.07, 0.03)	0.01 (−0.06, 0.08)	0.770
Relative fat intake (g/kg BW/day)						
Baseline	120	1.29 ± 0.46	117	1.27 ± 0.42		
12 weeks	115	1.25 ± 0.50	112	1.24 ± 0.48		
Within-group change ^c^	115	−0.03 (−0.10, 0.04)	112	−0.03 (−0.10, 0.04)	0.001 (−0.10, 0.10)	0.985
Relative carbohydrate intake (g/kg BW/day)						
Baseline	120	2.46 ± 0.99	117	2.33 ± 0.88		
12 weeks	115	2.42 ± 0.97	112	2.25 ± 0.78		
Within-group change ^c^	115	−0.04 (−0.15, 0.07)	112	−0.08 (−0.19, 0.03)	0.04 (−0.12, 0.19)	0.630
**Physical activity**						
Total physical activity (kJ/day)						
Baseline	121	2425 ± 1533	119	2194 ± 1636		
12-weeks	121	2322 ± 1936	119	2262 ± 1977		
Within-group change ^c^	121	−103 (−423, 217)	119	67 (−256, 390)	−170 (−625, 284)	0.463
Moderate to vigorous physical activity (kJ/day)						
Baseline	121	1538 ± 1288	119	1293 ± 1232		
12-weeks	121	1496 ± 1522	119	1421 ± 1451		
Within-group change ^c^	121	−42 (−286, 202)	119	128 (−118, 374)	−170 (−517, 176)	0.335

Baseline and 12-week data were estimated using descriptive statistics in Stata, with mean ± SD (or 95%CI) reported. Within-group changes and mean between-group differences for the change over time were estimated using linear mixed models with values representing means and 95%CI. ^a^ Difference in change between AAC + Ed vs. Control + Ed groups. ^b^ p-values for the interaction between treatment group and timepoint from linear mixed models. ^c^ Baseline-adjusted within-group change from post-estimation margins. ^*^ Indicate statistical significance within-group changes from baseline to 12 weeks (^***^p < 0.001, ^**^p < 0.01 and ^*^p < 0.05). Bold p-values indicate statistically significant differences. Abbreviations: AAC, abdominal aortic calcification; Ed, healthy lifestyle education; BW, body weight

### Additional analyses

In exploratory analyses within the AAC + Ed group only, baseline AAC24 scores were inversely correlated with changes in visceral adipose tissue mass (ρ = −0.1994, *p* = 0.037; Supplementary Table 1). Those with evidence of AAC had significantly greater declines in total fat mass (net difference in change [95% CI] −0.6 [−1.0, −0.1] kg, *p* = 0.016) and visceral adipose tissue (−31 [−61, −1] g, *p* = 0.044), compared to those without evidence of AAC (Supplementary Table 2). There were no associations between baseline AAC24 scores and BMI, body fat percentage, muscle strength, or physical function within the AAC + Ed group, and no between-group differences in those with evidence of AAC compared to those without evidence.

## Discussion

In this secondary analysis of the 12-week MODEL study, the provision of AAC imaging results with healthy lifestyle (diet and physical activity) education materials had no effect on body composition, muscle strength, or physical function, compared to education alone. This could be due in part to the provision of AAC not changing lifestyle behaviours in terms of relative dietary energy or macronutrient intake, or energy expenditure from physical activity during the 12-week study period. However, exploratory analyses within the AAC + Ed group demonstrated those with evidence of AAC at baseline had greater declines in certain measures of fat mass compared to those without evidence of AAC.

The finding that the provision of AAC imaging results with educational resources did not lead to changes in healthy lifestyle behaviours (diet and physical activity) compared to education alone [15] is likely why there were no effects on body composition and muscle health outcomes. A recent scoping review of previous RCTs investigating other types of vascular imaging to improve lifestyle and anthropometric measures reports negligible effects on BMI, body weight, and/or waist circumference [13], which is consistent with the overall lack of effect on body composition observed in the current study. A possible contributor to the lack of effect on both the primary study outcomes [15] and the measures presented in the current analysis, is that participants may not have clearly understood the vascular imaging results and their clinical importance, despite receiving specific education on AAC. Previous RCTs using carotid ultrasound or coronary artery calcification, which are more commonly used vascular imaging modalities in clinical practice, have been shown to be similarly ineffective for eliciting improvements in anthropometric outcomes over 1 to 4 years of follow-up [13]. Accurate perception of risk when providing subclinical or clinical cardiovascular risk information is essential to maximise the likelihood of older adults changing behaviour [26]. Therefore, patient-centred approaches that consider the health literacy and preferences of older adults are important to ensure understanding of the information presented [26]. The use of images to depict calcification and colour to depict risk in the current study, and in some previous RCTs investigating provision of vascular imaging results [13], aligns with qualitative evidence from health care consumers on their preferred format of absolute CVD risk presentation [27]. Overall, the current results add to evidence showing that provision of vascular imaging results with education may not be adequate to promote healthy lifestyle behaviour changes that can influence anthropometry, body composition and muscle health outcomes compared to education alone.

An important finding of the current analysis is the small but significant loss of total lean soft tissue mass (0.3–0.4 kg) within both groups, and loss of appendicular lean soft tissue mass (0.2 kg) in the AAC + Ed group, over 12 weeks. We previously reported a similar significant 0.6 kg decrease in body weight within each group [15], and the current analysis shows that lean soft tissue mass comprised at least half of this weight lost. Although not accompanied by declines in muscle strength or physical function, it highlights an important issue often observed with weight loss in older adults [28, 29]. Preserving muscle during weight loss is essential for older adults who are losing weight, to prevent consequential adverse outcomes that are consistently associated with poor muscle health [28, 30]. In the current study, the lean soft tissue mass loss observed likely relates to participants being provided with education resources targeted at cardiovascular health, but not specifically at weight loss or muscle health. Structured exercise, particularly progressive resistance training, is the most effective way to improve muscle health during ageing and can preserve muscle when completed during weight loss [31, 32]. While general physical activity guidance included in the education resources did encourage completing resistance training twice weekly as per national and global physical activity recommendations [19, 33], there was no structured exercise prescription, support, or equipment provided to participants. Adequate protein intake is also important to optimise muscle health, and to help preserve muscle during diet-induced weight loss [30, 34]. In the current study, the mean daily protein intake among participants was ~ 1.2–1.3 g/kg BW which is similar to what is recommended for older adults [35]. This suggests that adequate protein intake alone (in the absence of resistance training) is not effective to counteract weight-loss related muscle loss.

Our exploratory analyses suggest that AAC provision may help to reduce fat mass among those with (but not without) evidence of AAC, despite changes in most outcomes not being correlated with baseline AAC24 scores. This builds on our previously reported finding that weight loss was greater in those with evidence of AAC compared to without evidence (1.0 kg vs. 0.1 kg) [15]. It is also consistent with the Early Identification of Subclinical Atherosclerosis by Noninvasive Imaging Research (EISNER) RCT [36] which showed greater reductions in body weight over four years with increasing coronary artery calcification scores among overweight participants provided their results. However, within-group changes in the current study shows that approximately half of the weight lost by those with AAC in the current cohort was lean soft tissue mass. A difference in effect in those with and without evidence of AAC aligns with suggestions based on the Health Belief Model that behaviour change is more likely with greater perceived susceptibility and perceived severity of an adverse event [12, 37]. However, we did not see any differential effects on diet or physical activity behaviours between those with and without evidence of AAC [15], and the perception of risk among those with evidence of AAC is unknown. Overall, these exploratory results should be interpreted with caution, especially considering between-group differences by presence or absence of AAC were only observed for certain fat mass outcomes, and not for any muscle health outcome.

Strengths and limitations of the MODEL study have been reported in detail previously [15], but there are specific strengths of the current analysis. This is the first RCT to report effects of providing vascular imaging results with healthy lifestyle education on DXA-derived body composition and muscle health outcomes compared to education alone, building on previous literature reporting anthropometrical measures. However, there are several specific limitations that should be considered. Firstly, the education resources provided to both study groups were focused on cardiovascular health and cardio-protective lifestyle behaviours, so were not specifically designed to target body composition and muscle health outcomes. Second, the precision of DXA body composition assessment must be considered given that several of the significant body composition changes observed were of small magnitude. The precision error for total body fat and lean soft tissue mass and appendicular lean soft tissue mass is < 2% in our laboratory, based on repeated scans in a random sample of 30 individuals. DXA has also been shown to underestimate longitudinal changes in visceral adiposity compared with MRI-measured visceral adipose tissue [38]. Third, grip strength was the only objective measure of muscle strength or function. Additionally, the study may have been subject to healthy volunteer bias. Baseline BMI was 27.2 kg/m^2^, which is shown to be healthy for older adults despite being in the overweight category [39], and almost all participants had healthy lean soft tissue mass (95.4%) and grip strength (97.1%), based on current sex-specific sarcopenia cut-points [40]. Finally, analyses were not adjusted for multiple comparisons, however the likelihood of type I errors was minimal given the results were largely non-significant.

In conclusion, providing Australian older adults with AAC results in addition to cardiovascular and lifestyle education did not lead to improvements in their body composition or muscle health over a 12-week period, compared to education alone. While neither group experienced any marked changes in healthy lifestyle behaviours (e.g., diet or physical activity), there was a similar reduction in weight and lean soft tissue mass in both groups, which suggests lifestyle education alone is insufficient to achieve favourable body composition changes. Appropriately prescribed structured exercise training with nutritional support is needed to preserve muscle during weight loss. Tentative evidence suggests that providing AAC results may have a beneficial effect on certain fat mass outcomes in those who have evidence of AAC, with a greater perceived risk, but this is exploratory and requires further research.

## Supplementary Information

Below is the link to the electronic supplementary material.

Supplementary Material 1

## Data Availability

The data that support the findings of this study are subject to ethics approval and restrictions related to data being still collected as part of the 4-year follow-up study. Data may become available upon reasonable request to the corresponding author, subject to ethics approval once the follow-up study has been completed.
