# Supplementary material for: Body composition and muscle health changes after providing vascular imaging results in older adults: secondary analysis of a randomised controlled trial
Source: Aging Clin Exp Res. 2026 Feb 11;38(1):85. doi: 10.1007/s40520-026-03335-1 (PMC12960372; doi:10.1007/s40520-026-03335-1)
Supplement: Supplementary file 1 — Supplementary Material 1 [file 40520_2026_3335_MOESM1_ESM.docx]

**Supplementary Table 1.** Correlation between baseline AAC24 scores and 12-week change in body composition, muscle strength and subjective physical function outcomes within the AAC plus education (AAC+Ed) group.

|  | **AAC score** | |
| --- | --- | --- |
| **Change variables** | **ρ** | **p-value** |
| **Body composition** |  |  |
| BMI (kg/m^2^) | -0.1630 | 0.089 |
| Total fat mass ^a^ (kg) | -0.1604 | 0.094 |
| Body fat percentage ^a^ (%) | -0.1105 | 0.245 |
| Total lean soft tissue mass ^a^ (kg) | -0.1094 | 0.255 |
| Appendicular lean soft tissue mass (kg) | -0.1242 | 0.196 |
| Visceral adipose tissue mass (g) | **-0.1994** | **0.037** |
| **Muscle strength** |  |  |
| Grip strength (kg) | -0.0297 | 0.757 |
| **Subjective physical function (SF-36 questionnaire)** |  |  |
| Physical functioning | 0.0094 | 0.922 |
| Role limitations due to physical health | -0.0002 | 0.999 |
| Energy/fatigue | -0.0195 | 0.840 |
| Pain | -0.0451 | 0.639 |
| General health | -0.0503 | 0.601 |

Rho (ρ) is from Spearman’s correlation. Bold indicates a significant correlation between higher AAC score and greater change over 12 weeks. Abbreviations: AAC, abdominal aortic calcification; BMI, body mass index. ^a^ Head excluded.

**Supplementary Table 2.** Within and between-group changes in body composition, muscle strength and subjective physical function outcomes in AAC+Ed participants with and without evidence of AAC.

|  | **AAC (AAC24≥1)**  **n = 65** | **No AAC (AAC24=0)**  **n = 49** |  |  |
| --- | --- | --- | --- | --- |
|  | **Mean within-group change (95% CI)** | **Mean within-group change (95% CI)** | **Mean net difference in change (95% CI) ^a^** | **p-value ^b^** |
| **Body composition** |  |  |  |  |
| BMI (kg/m^2^) ^c^ | -0.2 (-0.4, 0.1) | -0.02 (-0.2, 0.1) | -0.2 (-0.5, 0.2) | 0.336 |
| Total fat mass ^d^ (kg) | -0.5 (-0.8, -0.2) | 0.1 (-0.3, 0.5) | **-0.6 (-1.0, -0.1)** | **0.016** |
| Body fat percentage ^d^ (%) | -0.2 (-0.5, 0.1) | 0.1 (-0.3, 0.6) | -0.3 (-0.8, 0.1) | 0.161 |
| Total lean soft tissue mass ^d^ (kg) | -0.6 (-0.9, -0.2) | -0.3 (-0.6, 0.05) | -0.3 (-0.8, 0.2) | 0.284 |
| Appendicular lean soft tissue mass (kg) | -0.3 (-0.5, -0.1) | -0.1 (-0.3, 0.04) | -0.2 (-0.5, 0.1) | 0.256 |
| Visceral adipose tissue mass (g) | -31 (-49, -13) | -0.001 (-26, 26) | **-31 (-61, -1)** | **0.044** |
| **Muscle strength ^c^** |  |  |  |  |
| Grip strength (kg) | -0.3 (-1.2, 0.5) | -0.1 (-0.9, 0.8) | -0.2 (-1.5, 1.0) | 0.690 |
| **Subjective physical function (SF-36 questionnaire) ^c^** | |  |  |  |
| Physical functioning | -0.3 (-3.4, 2.7) | 1.5 (-2.3, 5.3) | -1.8 (-6.6, 3.0) | 0.454 |
| Role limitations due to physical health | 2.3 (-8.1, 12.7) | 3.5 (-5.9, 12.9) | -1.2 (-15.5, 13.1) | 0.869 |
| Energy/fatigue | 1.1 (-1.8, 3.9) | 0.6 (-3.6, 4.8) | 0.5 (-4.4, 5.3) | 0.845 |
| Pain | 0.3 (-3.4, 4.1) | 2.1 (-2.4, 6.5) | -1.7 (-7.5, 4.0) | 0.549 |
| General health | 1.7 (-1.6, 5.0) | 3.8 (0.1, 7.5) | -2.1 (-7.0, 2.8) | 0.393 |

Data are means and 95%CI. ^a^ Difference in change between AAC vs No AAC participants. ^b^ p-values for the difference in change between groups from independent t-tests. ^c^ AAC: n = 65; No AAC: n = 50. ^d^ Head excluded. Bold indicates statistically significant between-group differences. Abbreviations: AAC, abdominal aortic calcification; BMI, body mass index.
